# Antitumor activity of combined endostatin and thymidine kinase gene therapy in C6 glioma models

**DOI:** 10.1002/cam4.798

**Published:** 2016-07-01

**Authors:** Yan Chen, Honglan Huang, Chunshan Yao, Fengbo Su, Wenming Guan, Shijun Yan, Zhaohui Ni

**Affiliations:** ^1^ Department of NeurosurgeryThe Second Hospital of Jilin UniversityChangchunJilin130021P. R. China; ^2^ Department of PathogenobiologyCollege of Basic Medical ScienceJilin UniversityChangchunJilin130021P. R. China

**Keywords:** C6 glioma, endostatin (ES), Ganciclovir, thymidine kinase (TK), tumor inhibition

## Abstract

The combination of Endostatin (ES) and Herpes Simplex Virus thymidine kinase (HSV‐TK) gene therapy is known to have antitumor activity in bladder cancer. The potential effect of ES and TK therapy in glioma has not yet been investigated. In this study, pTK‐internal ribosome entry site (IRES), pIRES‐ES, and pTK‐IRES‐ES plasmids were constructed; pIRES empty vector served as the negative control. The recombinant constructs were transfected into human umbilical vein endothelial cells (HUVECs) ECV304 and C6 rat glioma cell line. Ganciclovir (GCV) was used to induce cell death in transfected C6 cells. We found that ECV304 cells expressing either ES or TK‐ES showed reduced proliferation, decreased migration capacity, and increased apoptosis, as compared to untransfected cells or controls. pTK‐IRES‐ES/GCV or pTK‐IRES/GCV significantly suppressed cell proliferation and induced cell apoptosis in C6 cells, as compared to the control. In addition, the administration of pIRES‐ES, pTK‐IRES/GCV, or pTK‐IRES‐ES/GCV therapy improved animal activity and behavior; was associated with prolonged animal survival, and a lower microvessel density (MVD) value in tumor tissues of C6 glioma rats. In comparison to others, dual gene therapy in form of pTK‐IRES‐ES/GCV had a significant antitumor activity against C6 glioma. These findings indicate combined TK and ES gene therapy was associated with a superior antitumor efficacy as compared to single gene therapy in C6 glioma.

## Introduction

Glioma is the most common malignant tumor of the central nervous system 1. It is an aggressive tumor characterized by a high propensity for invasion and poor prognosis despite treatment with the currently recommended treatment comprising of surgical resection followed by adjuvant chemotherapy and radiotherapy 2. The average survival time for glioma patients is 12–15 months 3. Gene therapy refers to the introduction of a therapeutic gene or manipulation of a disease‐related gene, and is a promising approach to treatment of malignant glioma 4, 5.

Among all types of gene therapy (GT), suicide gene therapy 6 and antiangiogenic gene therapy 7 have proved to be effective against glioma tumor. Cancer is characterized by multiple gene mutations and disrupted molecular mechanisms 8. Therefore, gene therapy targeting single gene may have limited therapeutic outcomes, whereas combined (double) gene therapy may benefit from synergistic effects 4.

The most extensively studied suicide GT against glioma is herpes simplex virus thymidine kinase (HSV‐TK), which catalyzes the phosphorylation of nucleoside analog ganciclovir (GCV). Because of TK catalyzation, GCV can be converted into a toxic metabolite which inhibits DNA replication, cell proliferation and induces cell apoptosis 9, 10. Endostatin (ES) is an angiogenesis inhibitor which opposes antiglioma activities, with the most probable mechanism of restricting tumor microvasculature development 11, 12. The effect of combined TK and ES gene therapy on glioma has not yet been investigated.

In this study, we constructed recombinant plasmids expressing TK, ES alone or in combination, and analyzed their potential effects on cell proliferation, migration, and apoptosis of human umbilical vein endothelial cells (HUVECs) ECV304 and C6 rat glioma cell line. In addition, we determined the potential therapeutic effect of these recombinant plasmids in rats bearing C6 glioma tumor. Our findings may contribute to the development of high efficacy double‐gene‐targeted therapy against glioma.

## Materials and Methods

### Reagents

DMEM culture medium was obtained from GIBCO Inc., Grand Island, NY. Fetal bovine serum (FBS) was purchased from HyClone, Logan, UT. Anti‐CD34 and antiendostatin antibody were bought from Santa Cruz Biotechnology, Santa Cruz, CA. Annexin V‐FITC assay was obtained from Imgenex, San Diego, CA. Ganciclovir (GCV) was obtained from Roche, Basel, Switzerland.

### Construction of recombinant plasmids

The internal ribosome entrysite (IRES) vector was purchased from ClonTech, Mountain View, CA and the pCMV‐TK encoding the HSV‐tk gene was obtained from Dr. Li Chen the Department of Pharmacology, College of Basic Medical Sciences, Jilin University, China. Full‐length TK gene was amplified using pCMV‐TK as a template. Full‐length rat endostatin cDNA was amplified based on sequence provided by Genebank (GenBank accession No.: NM_009928). pTK‐IRES was constructed by cloning PCR‐amplified TK fragments into the NheI and EcoRI sites of pIRES vector. pIRES‐endostatin (ES) recombinant vector was constructed by inserting ES sequence at the restricted SalI and NotI sites of pIRES vector. pTK‐IRES‐ES recombinant plasmid expressing both TK and ES was constructed by inserting TK and ES sequences at the corresponding sites of pIRES vector as described in Fig.S1. The inserted sequence in all vectors was confirmed by DNA sequencing.

### Cell culture and transfection

Human umbilical vein endothelial ECV304 cells and rat C6 glioma cells were provided by Professor Liankun Sun the Department of Pathophysiology, College of Basic Medical Sciences, Jilin University, China. Cells were maintained in DMEM culture medium containing 10% FBS, and were cultured in a 5% CO_2_ incubator at 37°C. Furthermore, transfection was conducted when cells attained approximately 90% confluence. ECV304 cells were transfected with pIRES, pIRES‐ES, or pTK‐IRES‐ES plasmids, respectively, using Lipofectamine2000 transfection protocol according to manufacture's instructions (Invitrogen, Carlsbad, CA). Untransfected cells were used as normal control.

### Determination of the effects of recombinant plasmids on ECV304 cells

Forty‐eight hours after transfection, the mRNA and protein expression levels of ES were evaluated by reverse transcription polymerase chain reaction (RT‐PCR) and Western blotting, respectively. The influence of recombinant plasmid on cell proliferation was examined by MTT assay, and growth inhibition rate was calculated using the following equation:

$$\text{Growth}\;\text{inhibition rate}\;\left(\%\right)=\left(1-{\text{Absorbance}}_{\mathrm{Sample}}/{\text{Absorbance}}_{\mathrm{Control}}\right)\;\times \;100\%.$$

Cell migration capability was evaluated by wound healing assay and Transwell chamber system. Cell apoptosis was assessed by Annexin V/PI double staining followed with flow cytometric analysis (Becton−Dickinson Biosciences, Drive Franklin Lakes, New Jersey, USA). Data were obtained from three independent experiments.

### Determination of the effects of recombinant plasmids on C6 cells

Forty‐eight hours after transfection, the mRNA expression of TK was determined using RT‐PCR. In order to establish pTK‐ES/ganciclovir (GCV) system, single cell suspension of C6 cell lines was prepared and these cells were seeded onto 96‐well plate at a density of 1 × 10^5^ cells/mL in 100 *μ*L culture medium, and, transfection was performed on the next day. On day two after transfection, cells were exposed to different concentrations of GCV (0, 0.1, 1, 10 or 100 *μ*g/mL). The culture medium was changed after every 2 days. Three days after culture, cell morphology was examined under phase‐contrast microscope. Cell proliferation was determined by MTT assay. The ultrastructural changes were evaluated by transmission electron microscope. Cell cycle was determined by flow cytometric analysis 48 h after the initial GCV treatment. Three days after GCV exposure, cell apoptosis was evaluated by Hoechst 33258 staining and Annexin V/PI double staining followed with flow cytometric analysis. Data were obtained from three independent experiments.

### Establishment of rat C6 glioma and drug administration

All animal experiments were carried out in accordance with the Institutional Animal Care and Use Committee guidelines of Jilin University (Changchun, People's Republic of China). C6 glioma was produced in Wistar rats as previously described 13. Seven days after C6 cell inoculation, rats were examined by magnetic resonance imaging (MRI). After confirmation of the tumor mass, animals were randomly divided into five groups, each group comprising of nine animals. In normal control, empty vector control, pTK‐IRES/GCV, pIRES‐ES, and pTK‐IRES‐ES/GCV group, rats were given 15 *μ*L of PBS, pIRES, pTK‐IRES, pIRES‐ES, and pTK‐IRES‐ES by intratumoral injection under stereotaxic apparatus. Drug administration was repeated 7 days after initial injection. In pTK‐IRES/GCV and pTK‐IRES‐ES/GCV groups, animals received daily intraperitoneal injection of GCV at a dose of 50 mg/kg/d. GCV injection was administered 3 days post plasmid injection, and was continued for 8 days. Experimental procedures for animal studies are described in Fig.S2.

### Examination

The activity and behavior of animals in all experimental groups was monitored. MRI examination was carried out on Day 21 after tumor cell inoculation. The largest coronary and horizontal plane of the tumor was selected. The maximum length (L), width (W), height (H) was measured by MRI. The tumor size was calculated using the following equation: Tumor size (mm^3^) = n × L × W × H / 6.

Tumor growth inhibition rate was calculated using the following equation: tumor growth inhibition rate = (Control tumor size – Sample tumor size) / Control tumor size × 100%. Five rats were randomly selected from each group for analyzing survival time. The survival curve was drawn and animal survival was compared between different groups. Four animals in each group were used for histological examination. Briefly, on Day 22 after tumor cell inoculation, rats were anesthetized by intraperitoneal injection of 10% chloral hydrate. The rat brain was removed, fixed in 10% formaldehyde, and stained with hematoxylin–eosin (HE). Samples were immunostained with anti‐CD34 antibody using a SP kit as per the manufacturer's instructions (Maixin Biotech, Fuzhou, China), and the microvascular density (MVD) was measured according to a previously published method 14. In areas with most intense CD34‐positive neovascularization, micrographs were captured under ×200 magnification. Any endothelial cell or its cluster was considered as a single countable microvessel. The absolute number of the quantified microvessels per area was considered as the MVD value.

### Statistical analysis

Data were analyzed by SPSS 14.0 software (IBM SPSS New York, U.S.), and are expressed as mean ± standard deviation (SD). Statistical significance of intergroup differences was determined using analysis of variance (ANOVA) and *χ* ^2^ test. Animal survival was determined using Kaplan–Meier analysis and compare by means of the LogRank test. *P *<* *0.05 or *P *<* *0.01 was considered as significantly different.

## Results

### ES and TK‐ES inhibits the proliferation of ECV304 cells

**Figure 1:**
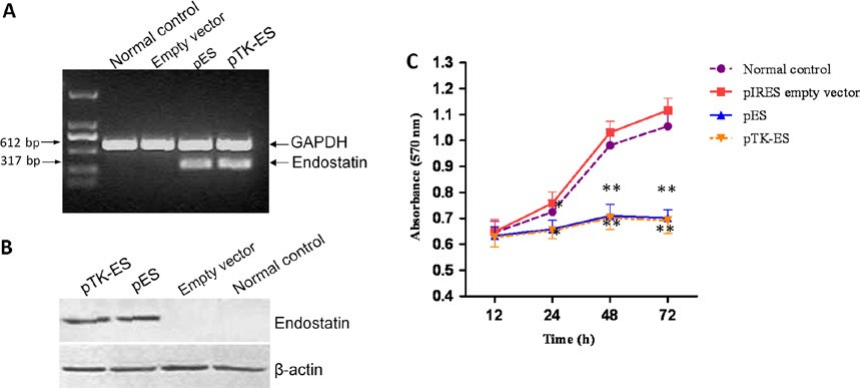
Endostatin (ES) and Thymidine kinase –Endostatin (TK‐ES) inhibited proliferation of ECV304 cells. Cells were transfected with pIRES (empty vector), pIRES‐ES or pTK – internal ribosome entry site(IRES)‐ES plasmids. Untransfected cells were used as normal control. Forty‐eight hours after transfection, the mRNA and protein expression of ES were evaluated by RT‐PCR (A) and Western blotting (B), respectively. GAPDH and *β*‐actin were used as internal controls. (C) The influence of recombinant plasmid on cell proliferation was examined by MTT assay. *N* = 3. * *P *<* *0.05, **P <* *0.01 compared with normal control. RT‐PCR, reverse transcription polymerase chain reaction; MTT, 3‐(4,5‐methylthiazol‐2‐yl)‐ 2,5‐diphenyltetrazolium bromide.

In order to determine the effects of pIRES‐ES and pTK‐IRES‐ES plasmids on ECV304 cells, these were transfected with pIRES empty vector, pIRES‐ES or pTK‐IRES‐ES plasmids. It was observed that mRNA and protein expression levels of ES were greatly upregulated in cells expressing ES and TK‐ES (Fig. 1A, B). In contrast, untransfected normal control or cells carrying empty vector did not express ES. In addition, there was a significant reduction in cell proliferation in cells expressing ES and TK‐ES at 72 h after transfection (*P *<* *0.01). pIRES‐ES and pTK‐IRES‐ES yielded a growth inhibition rate of 37.2% and 38.1%, respectively, at 72 h after transfection. No significant difference was observed with respect to the rate of cell proliferation between normal control and empty vector groups (*P *>* *0.05).

### ES and TK‐ES inhibits the migration of ECV304 cells

**Figure 2:**
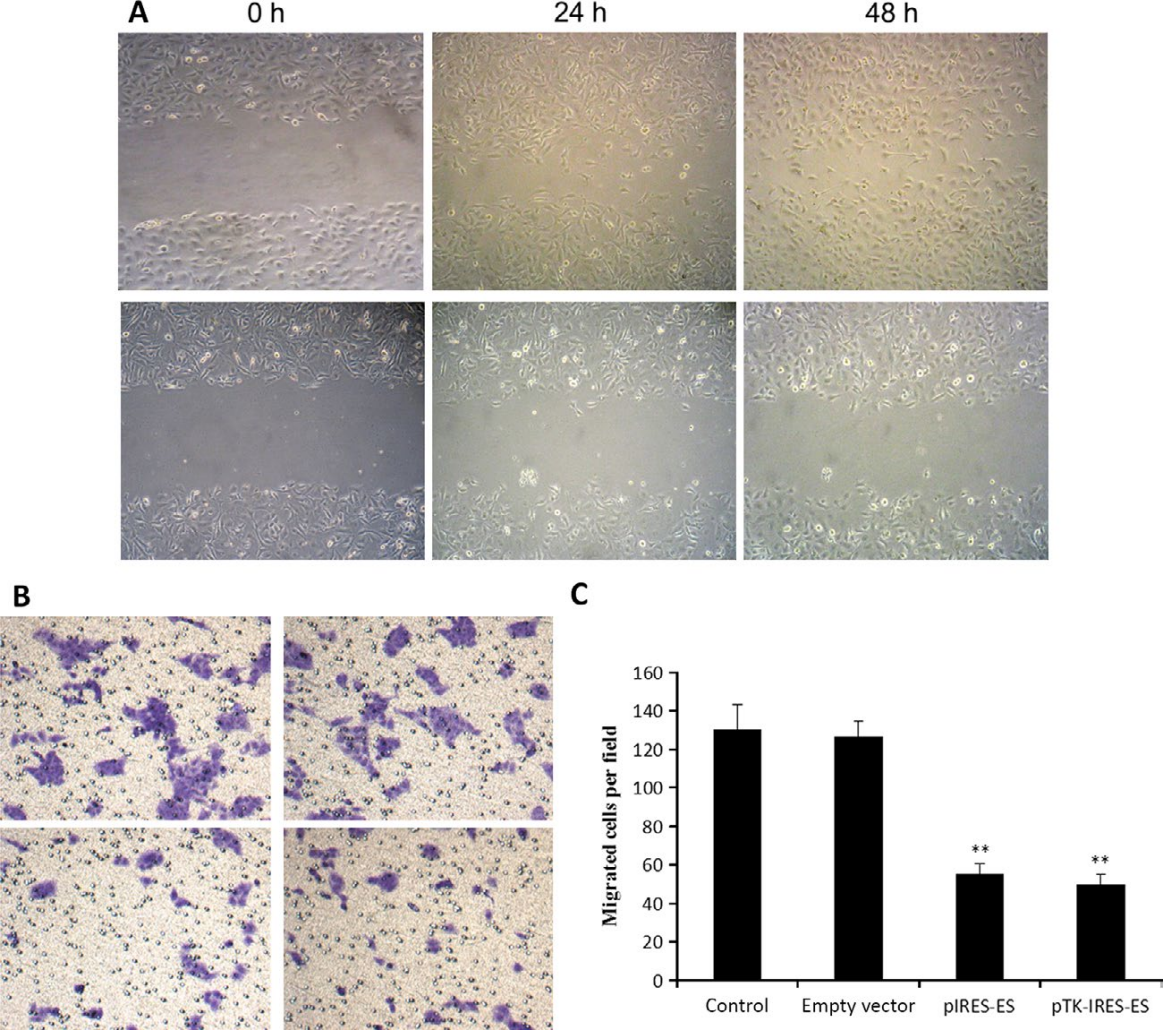
Endostatin (ES) and Thymidine kinase –Endostatin (TK‐ES) inhibited migration of ECV304 cells. (A) The extents of cell migration without (upper panel) and with (lower panel) inhibition evaluated by wound healing assay during 48 h. (B) The extents of cell migration for untransfected cells (upper left), pIRES transfected cells (upper right), pIRES‐ES transfected cells (lower left), and pTK–internal ribosome entry site(IRES)‐ES transfected cells (lower right) evaluated by Transwell chamber system. (C) Original magnification (×100) of cell migration for untransfected cells (control), pIREStransfected cells (empty vector), pIRES‐ES transfected cells (pIRE‐ES), and pTK‐IRES‐ES transfected cells (pTK‐IRES‐ES). *N* = 3. ** indicates *P* < 0.01 when compared with normal control group.

Cell migration capacity was determined by wound healing assay and Transwell chamber system. After scratching, the healing of the gap was completed within 48 h by cell migration toward the center of the gap (Fig. 2A). However, cells expressing ES or TK‐ES exhibited a decreased rate of “healing” of the gap, suggesting ES or TK‐ES may inhibit cell migration in ECV304 cells. This finding was confirmed by Transwell chamber system. The number of migrated cells was remarkably reduced in cells transfected with pIRES‐ES or pTK‐IRES‐ES as compared to that in the normal control or empty vector groups (*P *<* *0.01).

### ES and TK‐ES increases apoptosis of ECV304 cells

**Figure 3:**
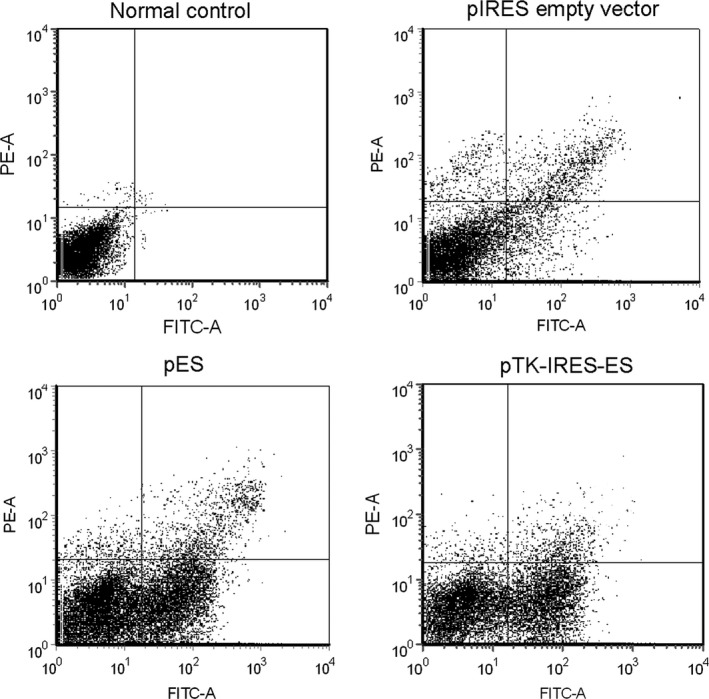
Endostatin(ES) and TK‐ES increased apoptosis of ECV304 cells. Cells were transfected with pIRES (empty vector), pIRES‐ES or pTK – internal ribosome entry site(IRES)‐ES plasmids. Untransfected cells were used as normal control. After 48 h transfection, cells were double labeled with Annexin V/PI and subjected for cytometric analysis. Cells were transfected with pIRES (empty vector), pIRES‐ES or pTK‐IRES‐ES plasmids show apoptotic rates of 12.4%, 31.4%, and 35.9%, respectively. The transfection of pIRES‐ES or pTK‐IRES‐ES significantly enhanced the percentage of apoptotic cells when compared with control. In addition, there was no significant differences in the number of apoptotic cells between empty vector and normal control groups (*P* > 0.05). *N* = 3.

Using Annexin V/PI double staining followed by flow cytometric analysis, we found that transfection of pIRES‐ES or pTK‐IRES‐ES significantly enhanced the percentage of apoptotic cells when compared with control (Fig. 3). It should be noted that no difference in the number of apoptotic cells between empty vector and normal control groups (*P *>* *0.05). The percentes of apoptotic rate were 12.4%, 31.4%, and 35.9% for empty vector, pIRES‐ES, pTK‐IRES‐ES experimental groups, respectively.

### TK/GCV and TK‐ES/GCV inhibits the proliferation of C6 cells

**Figure 4:**
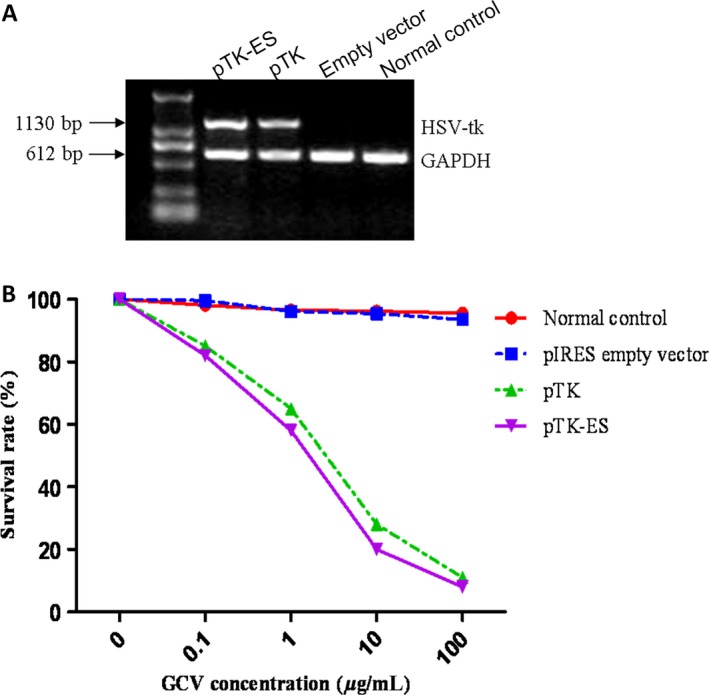
Thymidine kinase (TK) and Thymidine kinase –Endostatin (TK‐ES) inhibited proliferation of C6 cells. Cells were transfected with pIRES (empty vector), pTK – internal ribosome entry site(IRES) or pTK‐IRES‐ES plasmids. Untransfected cells were used as normal control. (A) After 48 h transfection, the mRNA expression of TK was evaluated by RT‐PCR, and GAPDH was used as an internal control. The mRNA expression of TK was greatly upregulated in cells expressing TK and TK‐ES, whereas untransfected normal control or cells carrying empty vector did not express TK. (B) With cells incubated with different concentrations of Ganciclovir(GCV), cell proliferation was examined by MTT assay after 3 days of GCV treatment. The survival rates in different groups showed that there was no significant cell growth inhibition for untransfected cells or cells carrying empty vector exposed to GCV, and there was a significant reduction in cell survival in cells those expressing TK or TK‐ES when administered increased doses of GCV. The survival rate of cells expressing TK or TK‐ES was only 11.2% and 8.3%, respectively, after exposure to GCV at a dose of 100 *μ*g/mL. *N* = 3. RT‐PCR, reverse transcription polymerase chain reaction.

To understand the effects of pTK‐IRES and pTK‐IRES‐ES plasmids on rat glioma cells, C6 cells were transfected with pIRES empty vector, pTK‐IRES or pTK‐IRES‐ES plasmids. The mRNA expression of TK was greatly upregulated in cells expressing TK and TK‐ES, whereas untransfected normal control or cells carrying empty vector did not express TK (Fig. 4A). Besides, when untransfected cells or cells carrying empty vector were exposed to GCV, no significant cell growth inhibition was observed (Fig. 4B). In contrast, there was reduced cell survival in those expressing TK or TK‐ES when administered increased doses of GCV. The survival rate of cells expressing TK or TK‐ES was only 11.2% and 8.3%, respectively, after exposure to GCV at a dose of 100 *μ*g/mL.

### TK‐ES/GCV induces S phase arrest and apoptosis in C6 cells

**Figure 5:**
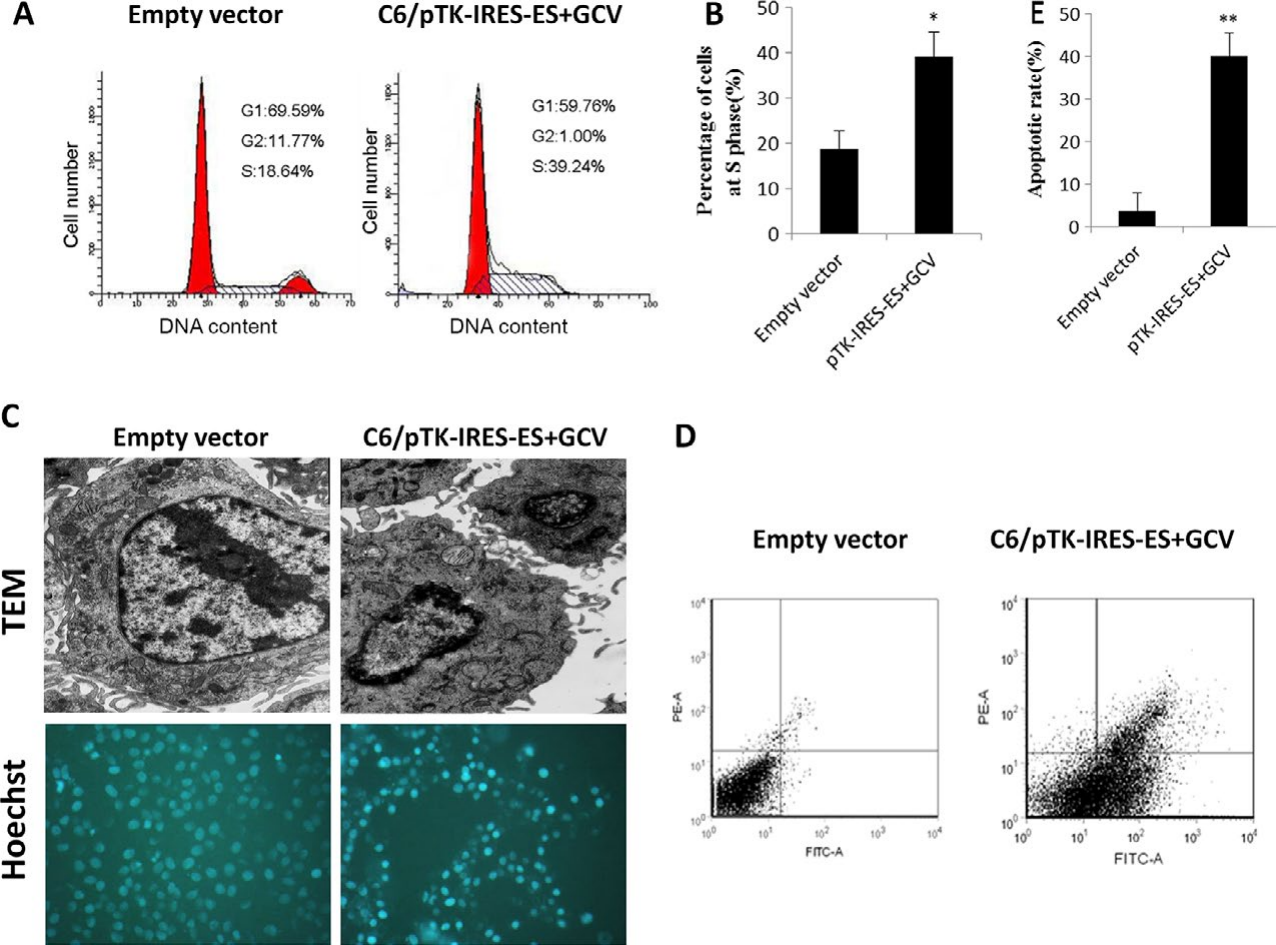
TK‐ES/ Ganciclovir(GCV) induces apoptosis in C6 cells. Cells were transfected with pIRES (empty vector control) or pTK‐IRES‐ES plasmids. Three days after treatment with Ganciclovir (A) Cell cycle was analyzed by flow cytometry. (B) The percentage of cells in S phase was determined. (C) The ultrastructural changes in cells were examined by transmission electron microscope. Cells were stained with Hoechst dye. *N* = 3. (D) Cell apoptosis was determined using Annexin V/PI double staining followed by flow cytometric analysis. IRES, internal ribosome entry site.

Flow cytometric analysis revealed that transfection of pTK‐IRES‐ES plasmids led to S phase cell arrest when incubated with GCV for 48 h. The percentage of cells in S phase was greatly increased in cells expressing TK‐ES when compared with empty vector control (Empty vector, 20.23%; pTK‐IRES‐ES, 39.24%) (Fig. 5A, B). However, the percentage of cells at G0/G1 or G2/M phases was reduced in cells expressing TK‐ES. Moreover, administration of GCV induced morphological changes in cells transfected with pTK‐IRES‐ES plasmids, observed as cell shrinkage and poor adherence. Electron microscopy revealed that normal control cells treated with GCV exhibited abundant microvilli, regular nucleus shape, clear nucleolus, and enriched intracellular organelles in cytosol (Fig. 5C). While cellular changes such as absence of microvilli, irregular nucleus, nuclear condensation, nuclear chromatin concentration and edge accumulation, mitochondrial cavitation, rough endoplasmic reticulum swelling as well as lysosomal accumulation were observed in cells expressing TK‐ES after GCV incubation. Hoechst 33258 staining further confirmed that cell apoptosis was indeed induced by TK‐ES/GCV (Fig. 5C). Besides, compared to normal control, TK‐ES/GCV resulted in a dramatical increase in percentage of early apoptotic cells (Empty vector, 3.8%; pTK‐IRES‐ES, 40.1%) as determined by Annexin V/PI double staining followed by flow cytometric analysis (Fig. 5D). These findings suggest that TK‐ES/GCV induced apoptosis in C6 cells.

### Therapeutic effects of recombinant plasmids in C6 glioma rats

**Figure 6:**
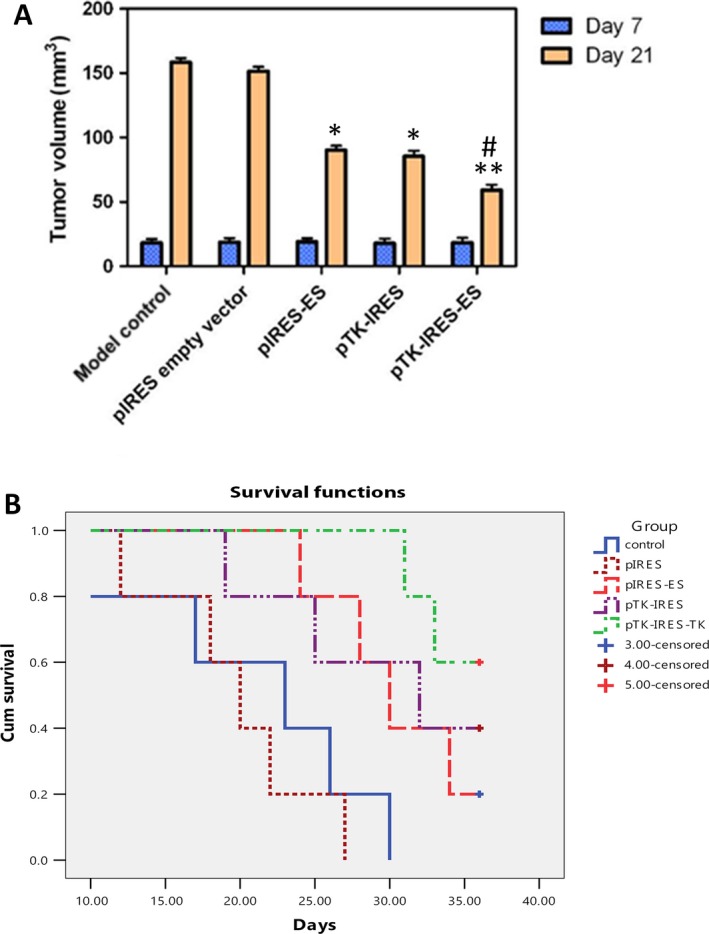
Tumor volumes and cumulative animal survivals for untransfected cells (control), pIRES transfected cells (empty vector), pIRES‐ES transfected cells (pIRE‐ES), pTK – internal ribosome entry site(IRES)‐ES transfected cells (pTK‐IRES‐ES) and pTK‐IRES‐ES transfected cells (pTK‐IRES‐ES). (A) Tumor volumes measured by magnetic resonance imaging (MRI) at 7th and 22nd Days of post‐C6 glioma cell inoculation. Recombinant plasmid therapy significantly inhibited tumor growth in C6 glioma rats. *N* = 5 for each experimental group. * indicates *P* < 0.05, ** indicates *P* < 0.01 compared with model control; # indicates *P* < 0.05 compared with either pIRES‐ES or pTK‐IRES. (B) Cumulative animal survival from Kaplan–Meier analysis was presented, and pTK‐IRES‐ES/ Ganciclovir(GCV) therapy appeared to be most efficient in inhibiting tumor cell growth.

**Figure 7:**
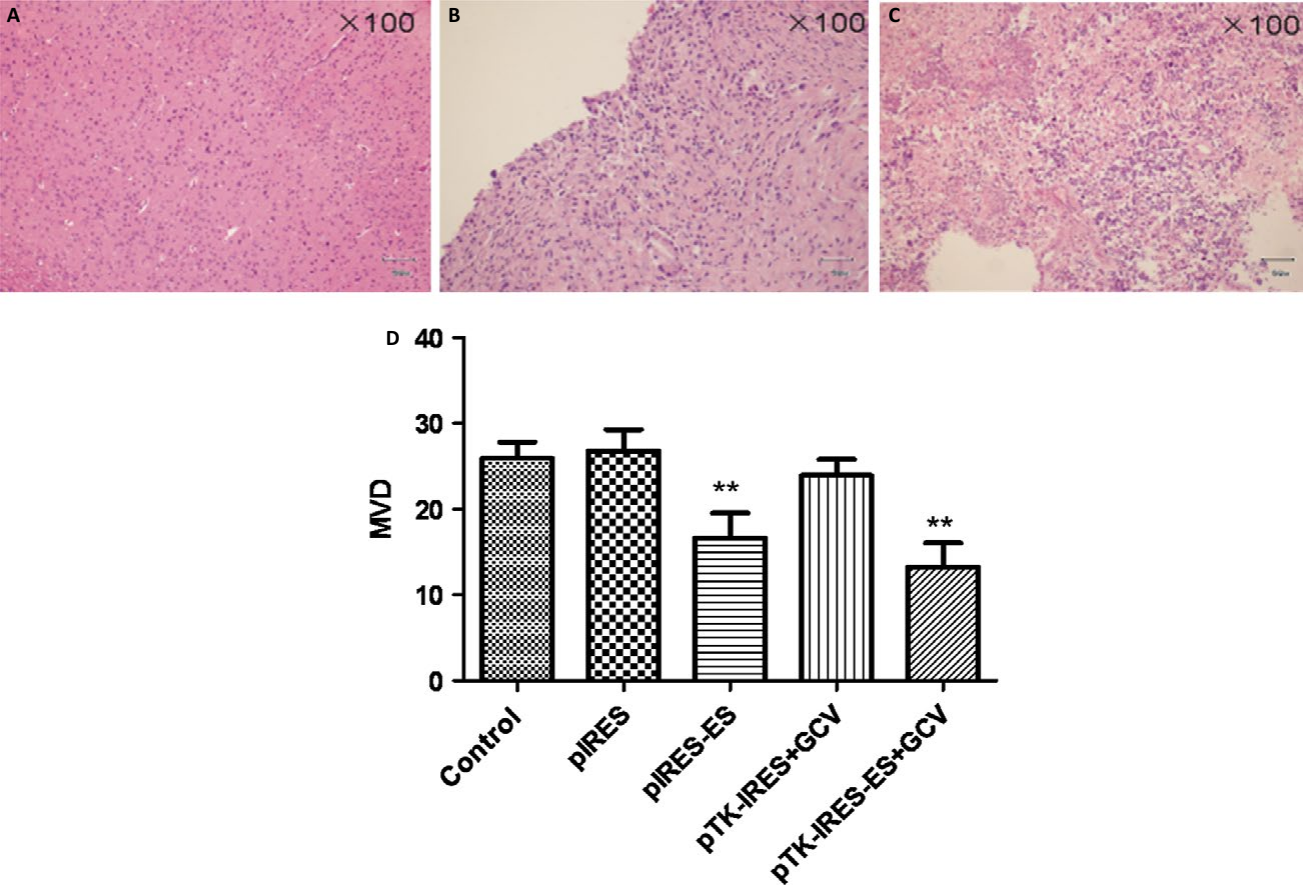
Histological analysis and microvessel density (MVD) values of rat tumor tissue by study group. (A) Histological examination of normal rat brain tissues, (B) tumors in model animals, (C).tumors in animals received pTK‐IRES‐ES treatment (D) MVD values of tumor tissues. **P <* *0.01 compared with control group. *N* = 4 for each group. MVD, microvessel density. IRES, internal ribosome entry site.

After 6 days of C6 glioma cell inoculation, rats showed decreased water and food intake, showed poor response, signs of mental fatigue, and occasional seizure attack, with reduced strength in left limb muscle, and unsteady walking. Recombinant plasmid therapy (pIRES‐ES, pTK‐IRES/GCV, or pTK‐IRES‐ES/GCV) improved the activity and behavior of animals bearing C6 glioma within 15 days of tumor cell inoculation. Moreover, recombinant plasmid therapy significantly inhibited tumor growth in C6 glioma rats (Fig. 6A). Of note, pTK‐IRES‐ES/GCV therapy appeared to be most efficient in inhibiting tumor cell growth (tumor growth inhibition rate: empty vector, 4%; pIRES‐ES, 43%; pTK‐IRES/GCV, 46%; pTK‐IRES‐ES/GCV, 63%; pTK‐IRES‐ES/GCV vs. empty vector, *P *<* *0.01; vs. pIRES‐ES or pTK‐IRES/GCV, *P *<* *0.05), and improving animal survival as determined by Kaplan–Meier analysis (Fig. 6B). Histological examination revealed that pTK‐IRES‐ES/GCV therapy resulted in an obvious cell morphological alteration and necrosis in tumor cells, as compared to that in the other groups (Fig. 7A). In addition, pIRES‐ES and pTK‐IRES‐ES/GCV therapy significantly reduced the MVD value of tumor tissues as compared to that of empty vector groups (*P *<* *0.01) (Fig. 7B).

## Discussion

The tumorigenesis and progression of malignant glioma involves complex mechanisms involving multiple genes. Combined gene therapy is a promising therapeutic approach to treatment of glioma 15. IRES‐based vector, which allows engineering of double‐ or triple‐gene constructs from a single mRNA, have been widely investigated in preclinical and clinical studies 16. In this study, we, for the first time, designed a double‐gene dicistronic expression constructs by inserting a suicide gene (TK) and an antiangiogenic gene (ES) at the appropriate sites of pIRES vector. In a eukaryotic system, TK and ES get transcribed from a single promoter in the same cell, which avoids the interferences of gene transcription from different promoters. Besides IRES‐based expression vector has many advantages over fused protein expression vectors, thus ensuring the sustenance of biological activities of TK and ES. Both RT‐PCR and Western blotting revealed expression of TK and ES in both ECV304 and rat C6 glioma cells.

The specific cells in the tumor microenvironment, such as endothelial cells, play a pivotal role in providing oxygen and nutrients for the expansion of tumor mass 17, 18. The crosstalk between glioma cells and host endothelial cells drives the progression of tumor 19, 20. Considering the close association between tumor cell and endothelial cells, it is proposed that deregulation of angiogenesis may benefit therapeutic outcomes of anticancer therapy when used in combination with surgery, chemotherapy as well as radiotherapy 21. ES is a potent angiogenesis inhibitor with low‐toxicity and a promising potential for clinical use 22, 23. ES has been shown to suppress the growth of glioma in animal models possibly by suppressing the development of tumor microvasculature 11, 12. In accordance with these findings, we detected that ES reduced proliferation, inhibited migration and induced apoptosis of ECV304 cells. In addition, administration of plasmid expressing ES gene also restricted tumor growth, reduced MDV, and prolonged animal survival in C6 glioma bearing rats. The molecular mechanism by which ES inhibits endothelial cell migration and induces cell apoptosis remains unclear. ES may contribute to reduced migration by modulating several factors, such as by inhibiting expression of integrin, matrixmetalloproteases (MMPs), and cytoskeletal protein tropomyosin 24. Besides, ES may trigger endothelial cell apoptosis through Shb adaptor protein‐dependent tyrosine kinase signal transduction pathway 25. However, the precise mechanism still needs to be investigated.

Despite the promising therapeutic value of antiangiogenic therapy in glioma patients, increased tumor metastasis is thought to result from an enhanced adaptive resistance 26. Therefore, combined use of antiangiogenic gene therapy and gene therapy directly targeting glioma cells may improve the therapeutic outcomes. In this study, HSV‐TK, the suicide gene most extensively tested in humans 27, was cloned into pIRES vector together with ES gene. To confer cytotoxicity, cells were transfected with plasmids encoding TK (TK alone or TK‐ES), followed by GCV incubation. The efficacy of TK/GCV system depends largely on the proportion of proliferating cells, in which TK/GCV inhibits DNA replication and cell proliferation 28. In our study, the percentage of TK‐expressing cells at S phase was greatly increased after GCV exposure, whereas the proportion of cells at G2/M phase was significantly reduced. Moreover, cell apoptosis was dramatically enhanced in TK‐expressing cells following GCV treatment. Hence, the antitumor activity of TK/GCV system might be related to its potency in causing S phase arrest and inducing cell apoptosis.

In our study, the in vivo results in rats with C6 glioma further confirmed the efficacy of combined gene therapy. We found that combined therapy greatly inhibited tumor growth, reduced MVD value in tumor tissues, improved disease symptoms and prolonged animal survival time as compared to single gene therapy. It is possible that TK‐mediated tumor cell inhibition may benefit ES‐modulated tumor microvasculature restriction, and thus lead to synergistic antitumor effects in C6 glioma rats. Our findings are consistent with the results of dual gene therapy for treatment of bladder cancer both in vitro and in vivo studies 29, 30, 31. The combined therapy yielded a significantly increased antitumor activity against bladder cancer when compared with single gene therapy 31.

Our results demonstrate the superior antitumor activity of combined TK and ES gene therapy, mediated, possibly, by inhibition of both angiogenesis and tumor growth. Combined gene therapy could prove to be a valuable treatment modality for malignant glioma.

## Conflicts of interest

None declared.

## Supporting information


**Figure S1.** Plasmid constructs.

**Figure S2.** Experimental procedures for animal studies. (A) Experimental procedure for animals in pTK‐IRES‐ES and pTK‐IRES groups. (B) Experimental procedures for animals in control (PBS), vector control (pIRES), and pIRES‐ES groups.

## References

[1] Omuro, A. , and L. M. DeAngelis . 2013 Glioblastoma and other malignant gliomas: a clinical review. JAMA 310:1842–1850. 24193082 10.1001/jama.2013.280319

[2] Chowdhary, M. M. , C. I. Ene , and D. L. Silbergeld . 2015 Treatment of Gliomas: how did we get here? Surg. Neurol. Int. 6:S85–S88. 25722937 10.4103/2152-7806.151348 PMC4338482

[3] Zahonero, C. , and P. Sanchez‐Gomez . 2014 EGFR‐dependent mechanisms in glioblastoma: towards a better therapeutic strategy. Cell. Mol. Life Sci. 71:3465–3488. 24671641 10.1007/s00018-014-1608-1 PMC11113227

[4] Okura, H. , C. A. Smith , and J. T. Rutka . 2014 Gene therapy for malignant glioma. Mol. Cell. Ther. 2:21. 26056588 10.1186/2052-8426-2-21 PMC4451964

[5] Bansal, K. , and H. H. Engelhard . 2000 Gene therapy for brain tumors. Curr. Oncol. Rep. 2:463–472. 11122879 10.1007/s11912-000-0067-z

[6] Tobias, A. , A. Ahmed , K. S. Moon , and M. S. Lesniak . 2013 The art of gene therapy for glioma: a review of the challenging road to the bedside. J. Neurol. Neurosurg. Psychiatry 84:213–222. 22993449 10.1136/jnnp-2012-302946 PMC3543505

[7] Gatson, N. N. , E. A. Chiocca , and B. Kaur . 2012 Anti‐angiogenic gene therapy in the treatment of malignant gliomas. Neurosci. Lett. 527:62–70. 22906922 10.1016/j.neulet.2012.08.001 PMC3471371

[8] Brat, D. J. , R. G. Verhaak , K. D. Aldape , W. K. Yung , S. R. Salama , L. A. Cooper , et al. 2015 Comprehensive, Integrative Genomic Analysis of Diffuse Lower‐Grade Gliomas. N. Engl. J. Med. 372:2481–2498. 26061751 10.1056/NEJMoa1402121 PMC4530011

[9] Moolten, F. L. 1986 Tumor chemosensitivity conferred by inserted herpes thymidine kinase genes: paradigm for a prospective cancer control strategy. Cancer Res. 46:5276–5281. 3019523

[10] Fischer, U. , S. Steffens , S. Frank , N. G. Rainov , K. Schulze‐Osthoff , and C. M. Kramm . 2005 Mechanisms of thymidine kinase/ganciclovir and cytosine deaminase/ 5‐fluorocytosine suicide gene therapy‐induced cell death in glioma cells. Oncogene 24:1231–1243. 15592511 10.1038/sj.onc.1208290

[11] Yang, L. , Z. Lin , J. Lin , S. Weng , Q. Huang , P. Zhang , et al. 2011 Antitumor effect of endostatin overexpressed in C6 glioma cells is associated with the down‐regulation of VEGF. Int. J. Oncol. 38:465–471. 21165557 10.3892/ijo.2010.871

[12] Zhang, G. , G. Jin , X. Nie , R. Mi , G. Zhu , W. Jia , et al. 2014 Enhanced antitumor efficacy of an oncolytic herpes simplex virus expressing an endostatin‐angiostatin fusion gene in human glioblastoma stem cell xenografts. PLoS ONE 9:e95872. 24755877 10.1371/journal.pone.0095872 PMC3995956

[13] Barth, R. F. , and B. Kaur . 2009 Rat brain tumor models in experimental neuro‐oncology: the C6, 9L, T9, RG2, F98, BT4C, RT‐2 and CNS‐1 gliomas. J. Neurooncol. 94:299–312. 19381449 10.1007/s11060-009-9875-7 PMC2730996

[14] Zhen, H. N. , X. Zhang , P. Z. Hu , T. T. Yang , Z. Fei , J. N. Zhang , et al. 2005 Survivin expression and its relation with proliferation, apoptosis, and angiogenesis in brain gliomas. Cancer 104:2775–2783. 16284993 10.1002/cncr.21490

[15] Candolfi, M. , K. M. Kroeger , A. K. Muhammad , K. Yagiz , C. Farrokhi , R. N. Pechnick , et al. 2009 Gene therapy for brain cancer: combination therapies provide enhanced efficacy and safety. Curr. Gene Ther. 9:409–421. 19860655 10.2174/156652309789753301 PMC2864138

[16] Renaud‐Gabardos, E. , F. Hantelys , F. Morfoisse , X. Chaufour , B. Garmy‐Susini , and A. C. Prats . 2015 Internal ribosome entry site‐based vectors for combined gene therapy. World J. Exp. Med. 5:11–20. 25699230 10.5493/wjem.v5.i1.11 PMC4308528

[17] Hanahan, D. , and R. A. Weinberg . 2011 Hallmarks of cancer: the next generation. Cell 144:646–674. 21376230 10.1016/j.cell.2011.02.013

[18] Casey, S. C. , M. Vaccari , F. Al‐Mulla , R. Al‐Temaimi , A. Amedei , M. H. Barcellos‐Hoff , et al. 2015 The effect of environmental chemicals on the tumor microenvironment. Carcinogenesis 36(Suppl 1):S160–S183. 26106136 10.1093/carcin/bgv035 PMC4565612

[19] Jeon, H. M. , S. H. Kim , X. Jin , J. B. Park , K. Joshi , I. Nakano , et al. 2014 Crosstalk between glioma‐initiating cells and endothelial cells drives tumor progression. Cancer Res. 74:4482–4492. 24962027 10.1158/0008-5472.CAN-13-1597 PMC4295931

[20] Charles, N. A. , E. C. Holland , R. Gilbertson , R. Glass , and H. Kettenmann . 2012 The brain tumor microenvironment. Glia 60:502–514. 22379614 10.1002/glia.21264

[21] Wang, Z. , C. Dabrosin , X. Yin , M. M. Fuster , A. Arreola , and W. K. Rathmell , et al. 2015 Broad targeting of angiogenesis for cancer prevention and therapy. Semin. Cancer Biol. 35:S224–S243. 25600295 10.1016/j.semcancer.2015.01.001 PMC4737670

[22] O'Reilly, M. S. , T. Boehm , Y. Shing , N. Fukai , G. Vasios , W. S. Lane , et al. 1997 Endostatin: an endogenous inhibitor of angiogenesis and tumor growth. Cell 88:277–285. 9008168 10.1016/s0092-8674(00)81848-6

[23] Persano, L. , M. Crescenzi , and S. Indraccolo . 2007 Anti‐angiogenic gene therapy of cancer: current status and future prospects. Mol. Aspects Med. 28:87–114. 17306361 10.1016/j.mam.2006.12.005

[24] Ferreras, M. , U. Felbor , T. Lenhard , B. R. Olsen , and J. Delaisse . 2000 Generation and degradation of human endostatin proteins by various proteinases. FEBS Lett. 486:247–251. 11119712 10.1016/s0014-5793(00)02249-3

[25] Dixelius, J. , H. Larsson , T. Sasaki , K. Holmqvist , L. Lu , A. Engstrom , et al. 2000 Endostatin‐induced tyrosine kinase signaling through the Shb adaptor protein regulates endothelial cell apoptosis. Blood 95:3403–3411. 10828022

[26] Lu, K. V. , J. P. Chang , C. A. Parachoniak , M. M. Pandika , M. K. Aghi , D. Meyronet , et al. 2012 VEGF inhibits tumor cell invasion and mesenchymal transition through a MET/VEGFR2 complex. Cancer Cell 22:21–35. 22789536 10.1016/j.ccr.2012.05.037 PMC4068350

[27] Greco, R. , G. Oliveira , M. T. Stanghellini , L. Vago , A. Bondanza , J. Peccatori , et al. 2015 Improving the safety of cell therapy with the TK‐suicide gene. Front. Pharmacol. 6:95. 25999859 10.3389/fphar.2015.00095 PMC4419602

[28] Wahlfors, T. , A. Karppinen , J. Janne , L. Alhonen , and J. Wahlfors . 2006 Polyamine depletion and cell cycle manipulation in combination with HSV thymidine kinase/ganciclovir cancer gene therapy. Int. J. Oncol. 28:1515–1522. 16685452

[29] Pan, J. G. , X. Zhou , G. W. Zeng , and R. F. Han . 2012 Potent antitumour activity of the combination of HSV‐TK and endostatin armed oncolytic adeno‐associated virus for bladder cancer in vitro and in vivo. J. Surg. Oncol. 105:249–257. 21953122 10.1002/jso.22107

[30] Pan, J. G. , R. Q. Luo , X. Zhou , R. F. Han , and G. W. Zeng . 2013 Potent antitumor activity of the combination of HSV‐TK and endostatin by adeno‐associated virus vector for bladder cancer in vivo. Clin. Lab. 59:1147–1158. 24273940 10.7754/clin.lab.2012.121109

[31] Pan, J. G. , R. Q. Luo , X. Zhou , R. F. Han , and G. W. Zeng . 2013 Suppression of bladder cancer growth by adeno‐associated virus vector‐mediated combination of HSV‐TK and endostatin in vitro. Clin. Lab. 59:1077–1089. 24273931 10.7754/clin.lab.2012.121021

