# Supplementary figures and images for: Antitumor activity of combined endostatin and thymidine kinase gene therapy in C6 glioma models
Source: Cancer Med. 2016 Jul 1;5(9):2477–86. doi: 10.1002/cam4.798 (PMC5055148; doi:10.1002/cam4.798)

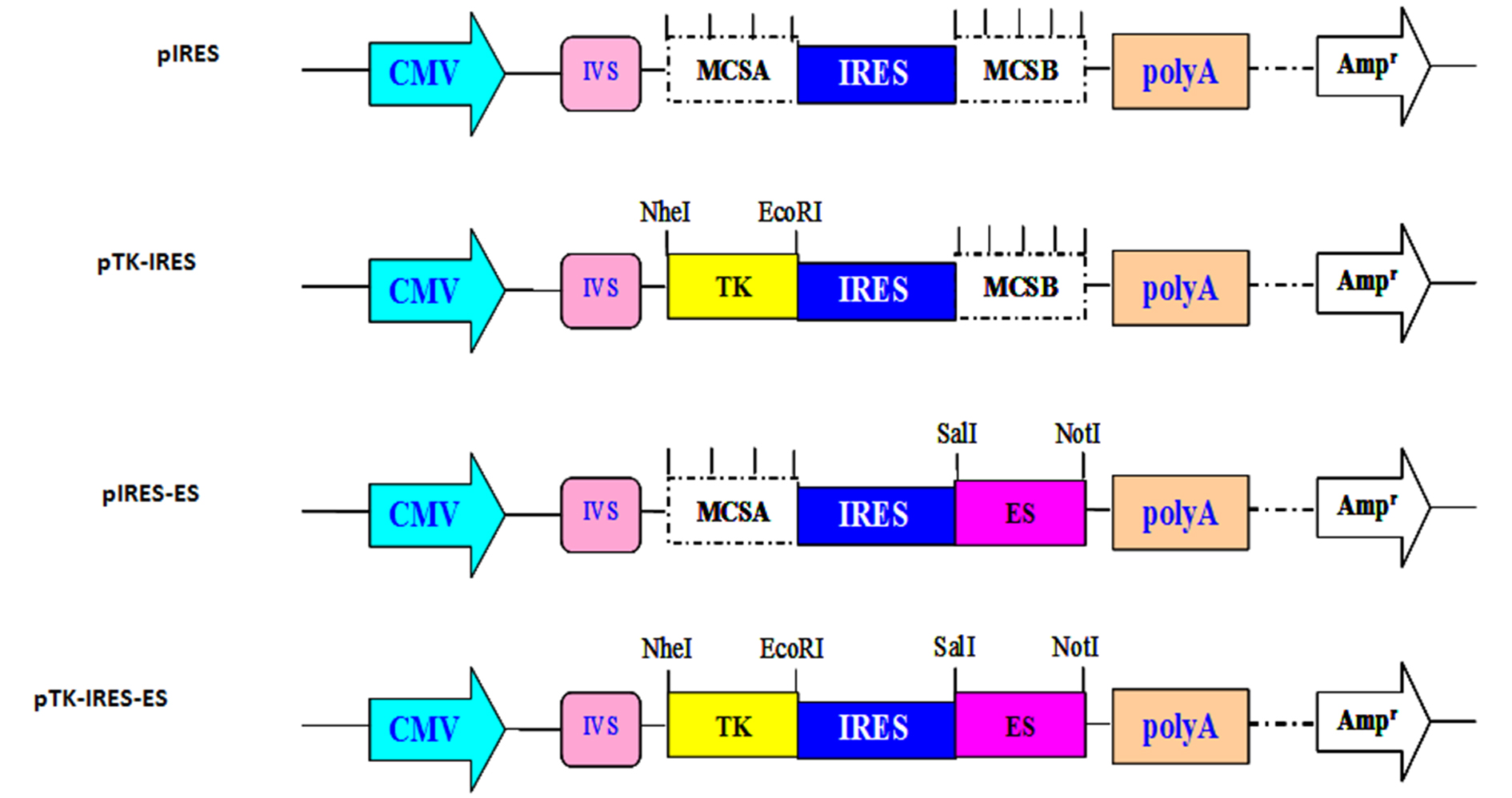

Supplement: Supplementary file 1 — Figure S1. Plasmid constructs. [file CAM4-5-2477-s001.tif]

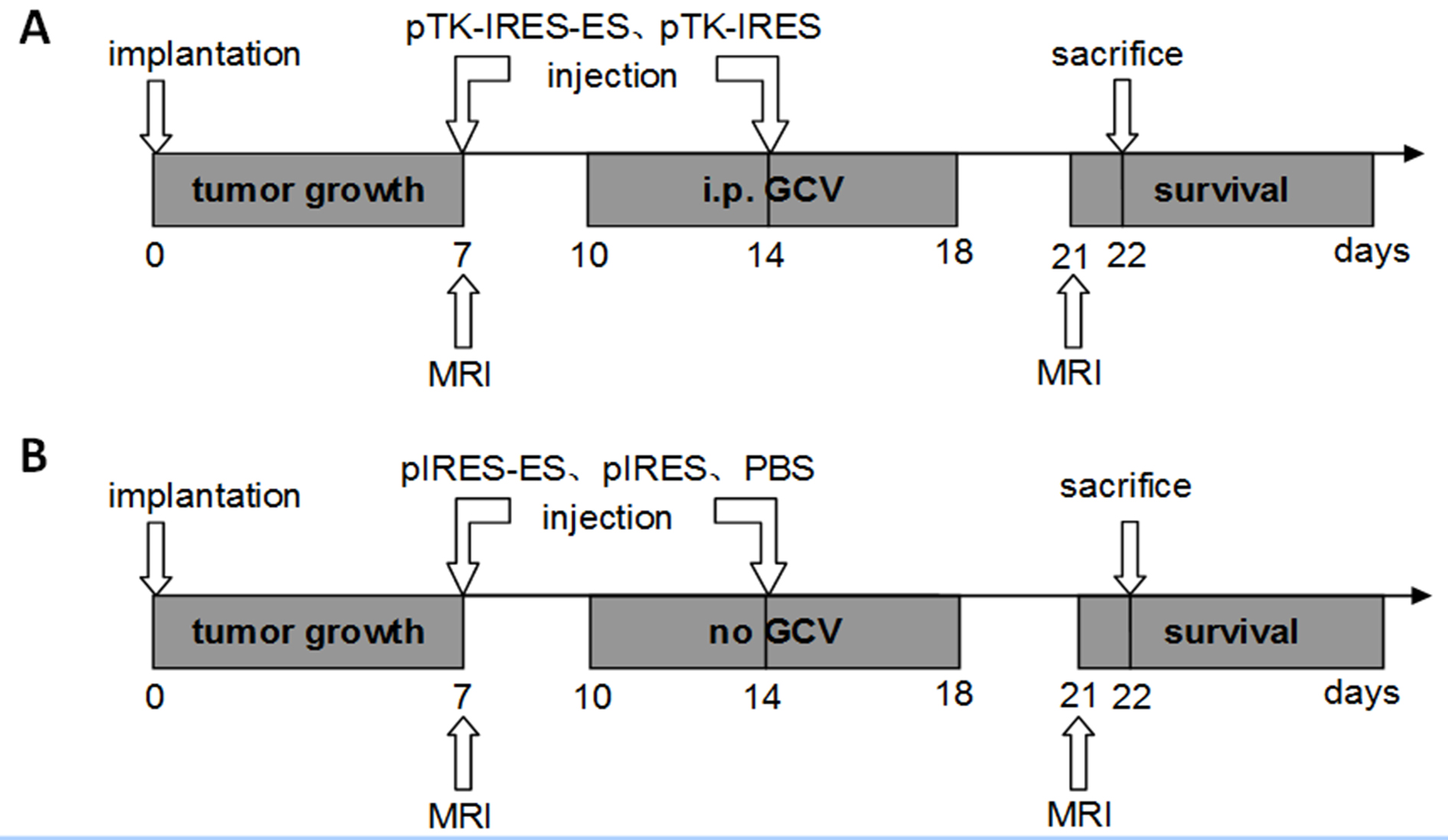

Supplement: Supplementary file 2 — Figure S2. Experimental procedures for animal studies. (A) Experimental procedure for animals in pTK‐IRES‐ES and pTK‐IRES groups. (B) Experimental procedures for animals in control (PBS), vector control (pIRES), and pIRES‐ES groups. [file CAM4-5-2477-s002.tif]
